# *The Ladder* by Eugenia Kuznetsova: A queer reading of a wartime Ukrainian novel

**DOI:** 10.12688/openreseurope.20792.1

**Published:** 2025-08-08

**Authors:** Eugenia Seleznova

**Affiliations:** 1 Department of Gender Studies, Central European University, Vienna, 1100, Austria

**Keywords:** contemporary Ukrainian literature, Russo-Ukrainian war, queer phenomenology, queer utopianism, spatiotemporality, gendered normativities, Ukrainian diaspora, Eugenia Kuznetsova

## Abstract

The article offers a queer phenomenological, after Sara Ahmed, and “queer world-making,” after José Muñoz, reading of the novel *The Ladder* ( *Драбина*) by Ukrainian author Eugenia Kuznetsova. The article examines gendered dynamics in the novel, and draws attention to its queer moments and features: more specifically, “competitive queer world-making” where the protagonist and his family appear as minoritarian subjects; “expanded space of the war” and changing spatiotemporal gendered normativities of Ukrainian citizenship and nationhood mediated through the gadgets, and multiplicity of queer phenomenologist “straight lines” affecting the protagonist. The paper also argues for the necessity of employing further gender and queer perspectives in the analysis of wartime Ukrainian fiction literature.

## Introduction

Despite the Russian full-scale invasion of Ukraine with all the obstacles it brings, Ukrainian fiction literature thrives, with multiple new books exploring diverse settings being published annually. Ukrainian authors who find strength and courage to write explicitly about the ongoing war gain the inevitable attention of the audience and critics.

Scholarship and criticism of Ukrainian wartime fiction literature are gradually developing (see 1– 3). However, there is a critical lack of gender and queer analyses of this literature. Given the role of gender and queerness in the political agenda surrounding the war ^4^ and the entanglements created by gendered Ukrainian martial law ^1^, it is particularly important to pay attention to how gender and sexuality are written in wartime Ukrainian fiction.

In this article, I present a recent popular Ukrainian novel, *The Ladder* ( *Драбина*) by Eugenia Kuznetsova, and analyse several gender- and queerness-related aspects that happened to be overlooked in previous reviews of the novel. By doing so, I aim to start covering the gap in gender and queer analysis of the recent wartime Ukrainian fiction and to investigate how the novel is reflective of the wider picture of reestablished gendered normativities and changing experiences of gender in wartime Ukrainian society.

## Introducing *The Ladder*: the plot and history of the novel’s publication

*The Ladder* is the second novel by Ukrainian author Eugenia Kuznetsova published in March 2023. It was awarded the “Book of the Year” prize by BBC Ukraine, one of the most prestigious and mainstream in the Ukrainian publishing industry.

The novel tells the story of Tolik ^2^, a Ukrainian-born IT specialist in his mid-to-late thirties, who put his best efforts into moving away from his judgemental and ever-intrusive family: mother, aunt, uncle, and sister. Tolik had made it as far as to Spain and had just bought his very first house, anticipating redesigning it to his taste. However, this was not meant to happen; just a few days after Tolik became a homeowner, his entire family arrived at his doorstep, escaping the war; his sister’s female friend, Polyna, tags along. Tolik did not have any option other than accommodating the whole group in his house; their incompatibility soon becoming the core conflict of the novel, developing concurrently with the ongoing war. Tolik’s masculinity was being particularly questioned: according to his family, he did not look fit and mature enough; had not mastered the handy crafts a proper man should have mastered, and did not have a girlfriend, which for them was indeed a problem. However, neither of these stopped them from relying extensively on Tolik’s help and addressing various requests. At some point, Tolik put a ladder outside of his window on the second floor so that he could simply reach his room uninterrupted by his family members’ requests, complaints, and comments. The act of climbing the ladder became central to depicting the character’s journey, as reflected in the novel’s title.

The novel has gained popularity among Ukrainian audiences and attracted several critical reviews. Ganna Uliura, in particular, praised the novel for addressing an “unspoken issue” of extended families having to endure the war under one roof ^5^. She also examined temporal relations presented in the book, Tolik and his relatives living in “different temporal modes,” Tolik having to work at night while everyone else is supposed to be asleep to avoid disruptions, and his family being profoundly attached to their “lost” past ^5^. Uliura’s and other reviews addressed the patriarchalism of Tolik’s family, the lack of characters’ progression throughout the story ^6, 5^, and kitsch being masterfully used as an artistic tool ^5– 7^. However, the reviews were made through a heteronormative prism and did not address the gender and sexual relations and normativities described and reproduced in the novel. I argue that it is important to employ gender and queer prism in wartime Ukrainian novels as it could be productive in discovering how gender and sexual normativities as well as their shifts occurring amidst the war are being reflected and, potentially, co-constituted in recent Ukrainian literature.

In this article, I explore several gendered and queer spatiotemporal moments written in the novel and how they are reflective of a wider picture of the reestablished gendered spatiotemporal normativities of Ukrainian nationhood during Russia’s full-scale invasion. I argue that *The Ladder*’s protagonist Tolik, while not being explicitly described as queer, follows queer patterns and is, in fact, a queer character who was, however, “straightened” (after Ahmed, see 8) by the constant presence of his family and reestablished gendered normativities of Ukrainian citizenship and nationhood mediated through online media and gadgets.

## Theoretical framework explained

For my analysis, I engage with two queer theoretical frameworks: queer phenomenology by Sara Ahmed and queer world-making and utopianism by José Muñoz.

In her “Queer Phenomenology,” Sara Ahmed describes how our gender and sexual identities and behaviours are being shaped through constant temporal and spatial interactions with our social surroundings and figures of authority — mainly by our families. She processes Husserl’s idea of orientation towards objects and arranging the objects within one’s “bodily horizons” according to one’s aimings to ask what *makes* a queer subject and *queer orientation*. Ahmed argues we are all pushed to be heterosexually oriented through what she calls “straight lines” — consequences of events and actions one is expected to undertake and reach for their life to count as “worthy” ^8^. These are such “milestones,” which enter heterosexual relationships, marriage, and childbirth. Through repetitive actions and life choices, an individual determines what is within their “bodily horizon,” and what is out of it: in the case of a “straight-oriented” person, these would be partners of the opposite sex ^8^. Various “straightening devices” are used to ensure an individual’s following the “straight lines,” from direct anti-queer violence to lovingly expressing concerns that one might not be happy as a queer (e.g. “a happy life is a heterosexual life, and I love you and want you to be happy”) ^8^.

Queerness then, according to Ahmed, emerges through repeating acts of “jumping off” a straight line, following the attachments and desires one develops towards other queer subjects accidentally occurring within one’s bodily horizons. One has to *become* queer by constantly making choices driving an individual further away from the “straight lines” drawn for them by predecessors. Apart from sexuality, Ahmed also examines how racialized bodies are being oriented and directed in spaces that are by default designed for white, heterosexual bodies; in these spaces, queer and racialized bodies are experiencing constant resistance, interruptions, delays, and discomfort, for these spaces are not “made” for them ^8^.

José Muñoz in his studies of performance and visual art has developed a complex theoretical framework describing how queer people of colour endure and resist racist heteronormative oppression by performatively and strategically subverting, “disassembling” mainstream narratives — disidentifying ^9^. He further argued that queerness is essentially utopianist, the future being “queerness’s domain,” bringing hope and something that is “always on the horizon” for the queers to aim for ^10^. In his analysis, Muñoz employed the term “queer world-making,” describing how “minoritarian performers” — queer artists of colour, in Muñoz’s studies, — were “establishing alternate views of the world” through their “disidentificatory” performances, “both theatrical and everyday rituals” ( 9 on page 195). The aim of such “world-making,” according to Muñoz, was not simply to “offer additional perspectives,” but to actively “deform and re-form the world,” and destabilise “hegemonic world visions,” so that the world would eventually become a better place for those “minoritised” ( 9 on page 196).

In my analysis of *The Ladder*, I use theoretical tools offered by Ahmed and Muñoz to see how the novel depicts Ukrainians’ gendered spatiotemporal experiences affected by the changing wartime normativities of nationhood and citizenship. Both authors indeed developed their concepts in a context quite different from the one *The Ladder* navigates; therefore, I have to apply them as “travelling concepts” ^11^.

My understanding of queerness also partially relies on the strand of scholarship taking this notion beyond merely sexuality and gender (non)normativity frames — for instance, that of Kalindi Vora and Neda Atanasoski, arguing that the post-socialist temporalities are “queer” — non-linear, nostalgic over the past, evading linear progress ^3^ — as opposed to the neoliberal Western temporalities aiming for “teleological” progress ^12^ ^4^. Further, Sima Shaksari argues, on the example of Iran under the Western sanctions, that the whole nations can be “queered” as not fitting the neoliberal Western standards and marginalised by the dominant geopolitical powers (in Shakhsari’s example, those of the West) even if the inner politics of the states under question are harshly homophobic ^13^. While the Ukrainian case is different by so many means, I find the way Ukrainian refugees from 2022 did not fall under numerous European normativities — or, following Ahmed, “straight lines” — to be a somewhat similar example of “the nation being queered” regardless of individual sexual behaviours. While heteronormativity is the focus of my analysis, I am also inquiring into how this “geopolitical,” non-sexual sort of queerness is present in *The Ladder*.

## Shades of queerness in *The Ladder*’s “competitive queer world-making”

The novel’s plot is largely kept in motion by the acts of spatial rearrangement conducted by the characters. First, Tolik buys the house he aims to redecorate. The spaces he had inhabited before were always marked by someone else’s gendered presence, and he always had to oblige someone else’s rules. First, his family’s home, where he had to share the room with his sister, was divided only with a pink “Japanese Sakura” screen the sister had chosen ( 14 on page 19). Then, the dormitories of his universities in Ukraine and further abroad — always crowded by other male students with “typically male” habits such as constantly watching football and inviting girls ( 14 on page 19–20). Finally, the apartment Tolik co-rented with Raul, a “sad man” approaching middle age, always listening to heavy metal music, and dreaming of a “cool car.”

When Tolik finally has the means to buy his own house, he does not look for one already corresponding to his liking. Instead, he purchases a house that is deeply marked by traces of the previous owner’s presence: an elderly, profoundly religious Spanish woman, a “matriarch” to an extended family. Tolik hopes to convert this place into his own utopian “nest”:

An office, a cellar with a pinball machine, a bedroom with a plasma TV and a Playstation, a comic book library covering the entire wall, a bathroom with remotely controlled lights along the ceiling, a terrace with an armchair and a garland of solar lanterns, yellow pillows, and abstractionist carpet… ( 14 on page 19) ^5^
Tolik imagined his kingdom with smart lights, coffee machines, and games so often that the dream of a house has turned into his life mission. When he finally had enough money, Tolik started house hunting… He didn’t want a ready one, because the ready one that he wanted didn’t exist. So Tolik was looking for something to turn into a house of his dreams. The mere act of the transformation, turning a stranger’s box into his own kingdom was meant to be a separate delight. ( 14 on page 20–21)
It is, indeed, of particular importance for Tolik that the house of his dreams is “exactly four thousand kilometres away from home”, so that “his mother will not bring a basket of cherries, make him run to the market to buy apricots for the jam, and won’t even present him the blanket with deers she’s been keeping for Tolik for a while. Let Irusya [Tolik’s sister] have the blanket”. ( 14 on page 20)

Tolik begins getting rid of the previous owner’s heritage — ceramic Jesuses and angels’ figurines, embroideries with her children’s and grandchildren’s names covering the walls — when his family arrives. While Tolik’s space-transforming practices are on hold, as he is busy accommodating them, the family members themselves begin attempts to reshape his space. His aunt, Hryhorivna ^6^, announced that the fountain bowl in the backyard should be turned into a tomato seedbed, contrary to Tolik’s plan to restore the fountain. She wishes to grow the tomatoes herself, as she despises those Tolik brings from the local Spanish supermarkets — “they look beautiful, but they don’t even smell tomatoes” ( 14 on page 48). Tolik’s mother, on the other hand, familiarises herself with the new space through an act of cooking, more particularly — kneading the dough. Very soon, the dough was everywhere, “rising, gurgling, hissing and sputtering” ( 14 on page 23), “haunting” Tolik from every corner.

Eventually, Tolik finds his whole house turning into a territory of hierarchies and disciplining, himself being at the bottom of the “power ladder” despite owning the place. He physically struggles to reach his room on the upper floor, as someone would always stop him from requesting something or commenting on his appearance, habits, skills, etc. Many of these comments are related to Tolik’s apparent lack of masculinity: the elders are worried that he is “too thin,” “going to destroy his knees with running,” or even acquire erectile dysfunction and infertility due to caffeine consumption ( 14 on page 63–64). The fact that he does not have a girlfriend is also a frequent topic of discussion, to Tolik’s sincere incomprehension of how he is expected to “manage” to get himself a girlfriend on purpose.

When Tolik’s attempts to verbally defend his boundaries fail, he retreats to simply avoiding contact and rerouting his movement around the house. He buys a ladder and places it near the rear wall of the house so that he can climb into the window of his room without meeting anyone. He feels ashamed and embarrassed for having to “sneak like a criminal into his own window” ( 14 on page 100), yet the ladder becomes a point of relief: he sometimes feels the urge to get back to the ladder even when he is already in his room, just listening to his relatives downstairs yelling out the news of further Russian attacks ( 14 on page 101). As the ladder was standing in the bougainvillaea bushes growing behind the house, Tolik would often get physical traces of his path — little flowers would get stuck in his leg hair. His involuntary guests learned about the ladder soon, yet everyone chose not to speak about it and “pretend it was Tolik’s secret” ( 14 on page 103).

Reading *The Ladder* through Ahmed’s queer phenomenology and Muñoz’s queer utopianism prisms, I came to the conclusion that, first, “competitive spatial worldmaking” ^9^ takes place in the novel. Tolik aims to create his own little utopia in his new house, where nothing reminds him of his heteropatriarchal family. As soon as they are forcibly reunited under his roof, his relatives start employing “straightening devices” ^8^, verbally disciplining Tolik but also trying to prevent him from “moving wrong” within the space — e.g. from running out for a jog which apparently is going to make Tolik’s already “too thin” (for a proper male) legs even thinner. They are also rearranging Tolik’s space — not as much to “straighten” him but rather attempting to restore the lost utopias of their Ukrainian households and ways of life. Tolik’s relatives are “minoritised” ^9^ in their own ways — displaced, not fitting any of their new surroundings and not wanting to “stay and adapt” either; lacking understanding from people around due to language as well as political barriers, surrounding Europeans being way too sympathetic towards “simple and nice Russians”. These older Ukrainian refugees are queer as well — just not in terms of sexual identity and orientations, but in the “older” sense of the word: odd, “out of place” ( 8 on page 179). Their case is representative of what was happening to Ukrainian refugees on a larger scale when arriving in Europe in 2022 and urges for deeper investigation into what constitutes a queer “minoritarian subject” ^9^ in nowadays neoliberal, oftentimes homonormative ^17^ if not homonationalist ^18^ Western European societies ^7^. 

My second conclusion is that the very way Tolik experiences constant interruptions while moving through his own house and how he solves it by inventing an alternative route renders him queer, albeit his queerness never being explicitly stated in the novel. Tolik tries to escape “the straightening devices” of his family and goes a “queer route” with the ladder — an odd and indirect one. It makes him ashamed; it leaves traces; his family chooses to ignore it: a pattern familiar to many of those who had once “dropped the straight line” and “became” queer ^8^.

## Expanded space of the war mediated by gadgets

Current feminist war and conflict studies scholarship disproves the binaristic spatial notion of war and peace as strictly localised and existing separately, so that “there are places where there is war, and there are places where there is peace” (see 21; 22). Instead, the concept of the “everywhere war” ^23^ is gaining increased attention and development, defining the war as transgressing merely the locations of physical fights and attacks, and impacting communities across the globe, thousands of kilometres away from where the bombs are being dropped ^23^. Several authors pay particular attention to how bodies and bodily experiences of the war “serve” to expand it through time and space: for instance, Johanna Mannegren Selimovic has studied how those forcibly displaced endure the “streams of violence”, starting in their homelands with the conflicts leading to their displacement, and continuing with violence they have to endure as “unwanted” immigrants ^21, 24^.

Nataliia Otrischenko in her study of alteration of time perception in Ukrainians affected by the war argues that the Russian full-scale invasion of Ukraine has imposed on Ukrainians “violent expansion of the present”, striping them of access to their future plans and perspectives as well as to the past accumulated in belongings, familiar surroundings, and social statuses and communities acquired over prolonged periods of time ^25^. I argue that *The Ladder* provides an accurate depiction of how perceptions of space and spatiality for those affected were also altered by the war; namely, the “space of the war” is being as violently expanded as the “time of the war” ^25^. However, unlike the above-mentioned studies where bodies and sensual experiences of the war victims are the described as main “transmitters” of the war space ^21, 24^, in *The Ladder* we observe the war spatially expanding itself through gadgets and online media.

Despite being in immediate physical safety, the characters of *The Ladder* cannot escape the fact that the places and spaces they used to think of as their own are being destroyed, the destruction “aired live” through multiple media channels. Witnessing this destruction even from afar hurts, one cannot easily detach themselves from this pain. They are also aware that at any moment someone they love who is staying in Ukraine can be hurt or killed — as it ultimately happens to Hryhorivna’s niece, Tolik’s cousin, Slavka. The whole family is constantly “glued” to their gadgets, where the news of Russians’ attacks, strikes and casualties are flowing non-stop. Hryhorivna is also permanently on the phone with her girlfriends, an “intelligence network covering the whole of Ukraine” ( 14 on page 43) who are updating her about the “shellings and horrors of occupation” ( 14 on page 43). The war thus inhabits the living room, even though actual battles are happening on the other side of the continent. The bodies are safe from missiles but not from constant information flows, news and messages filling the guts with panic, anger, and grief.

*The Ladder* gives us an example of how the space of war is being expanded and spread digitally, processed by a gadget before capturing the bodies and minds of those affected, and producing corresponding emotions and bodily reactions to be carried further. The ability to perceive this extension and its consequences, and to share this violent experience, becomes a strong marker of belonging. Tolik realises that, however, alienated he feels from his relatives, and the war reaching their guts through the phone screen constitutes for them more commonality than he will ever have with any of his Western counterparts.

For the first time during the war, Tolik went downtown and stared at people with some sort of incomprehension: none of them has relatives with destroyed houses at home, none of them is afraid to glimpse at their phones. ( 14 on page 41)

Tolik’s sister, Irusya, and Polina, her friend who came from almost entirely occupied Kherson oblast,’ shared similar feelings and observations soon after the “charitable party for Ukraine” organised by Tolik’s colleagues:

— That all seems so distant right now. Like that party — as if it is a parallel world, — she [Irusya] said. — I hated all those “not ours” there, you know why?
— Why?
— Because their smartphones aren’t a gateway to hell. Imagine, they just take their phones — and stare at them calmly. They don’t get hit by panic in those three seconds while the news site is uploading.
— You know, I will never be able to grab my phone again without thinking about the chthonic horror that might arrive from there, — replied Polina. She was sitting on the bed and looking at her friend in the mirror.
— A black brick that is always ready to attack you, — said Irusya, bringing her face closer to the mirror, as if looking for something in her own eyes.
— Maybe this is what they meant by the rebellion of the machines?
Before finishing the phrase, Polina already had the phone in her hands again, its screen flashing headlines, multiplying the dreads and horrors of all these days. ( 14 on page 164)

Some of the characters try to utilise this expanded spatiality in reverse to contribute to the fight from afar. Irusya has volunteering “shifts” when she spends time “answering the comments on Instagram.” ^8^ Tolik, being particularly enraged and disappointed during the party by his conversations with Western colleagues, who kept bringing up examples of the “good, nice Russians they knew,” feels better once he donates a bit for the Ukrainian Armed Forces:

He grabbed the third cocktail and drank again before going to the cellar. He opened the air raid alarms app — the whole map was red. “Targeted strikes, my ass!” — he muttered. In the toilet stall, he opened the banking app and threw some money to a charity account — the first one that mentioned weapons. He felt like donating for the biggest bomb in the world, the one that would fall on Moscow directly. Only once the virtual banking assistant confirmed the payment, Tolik could breathe a bit easier for a moment. ( 14 on page 119)

However, contributions from afar were clearly not valued. Irusya’s “shifts” are disregarded by the family elder Anatolii Stepanovych. Tolik himself constantly feels ashamed, “a loser” for not being physically in Ukraine and not fighting Russians directly, especially after his cousin gets killed on the battlefield. This shame is indicative of Tolik’s diversion from the wartime gendered spatiotemporal normativity that dictates that all male citizens remain inside Ukraine, return from abroad, if needed, and head out to the battlefield. Constantly upcoming news of further Russian attacks deepens his shame for “not being there, not fighting,” and thus mediates the change in normativity.

Further exploration of how Ukrainians’ spatial perceptions are altered during the war and mediated by various gadgets and media is necessary, as well as defining the role of the spatial component in reestablished normativities of citizenship and nationhood for Ukrainians. It is already clear that being in- or outside of Ukraine during Russia’s full-scale invasion constitutes “a split” between the social groups (Osypchuk, cited in 26), particularly in the case of male Ukrainian citizens, the majority of whom are legally restricted from leaving the country ^27^. Looking beyond the strictly binary notion of “there – not there” and studying different spatial forms of “in-betweenness,” including those digitally mediated, is crucial for developing a more complex, non-essentialist understanding of spatial experiences and reestablished spatial normativities affecting Ukrainian individuals during the war.

## “Straight lines” divided, taking over

In queer phenomenological reading, *The Ladder* is a novel about the “straight line” and “straightening devices,” eventually taking over a queer protagonist. At some point Tolik “comes to terms” with his family’s ever-intrusive presence and constant disciplining; he figures out he belongs to them more than to his European counterparts despite what he believed to be insurmountable differences. He even fulfils his family’s long-term wish and “finds” himself a girlfriend, by following his relatives’ multiple hints and entering heteronormative relationships with Polyna, his sister’s friend. As Polyna considers returning to Ukraine by the car bought by Tolik’s colleagues for the Ukrainian Armed Forces with fundraised money, Tolik joins her. Finally, Tolik breaks the ladder apart, so that he never has to enter his house “the queer” way again — only through the front doors like everyone else does. He no longer wishes for his relatives to leave, contrary to his eagerness to escape them and the “straight lines” they embodied in the first half of the novel. However, they leave as soon as it becomes possible once the Kyiv oblast’ is deoccupied.

Muñoz warns us that disidentificatory practices have their own limits, and that the transformations they were supposed to bring can fail ( 9 on page 161–162). The novel tells very little about what happens to Tolik after he enters into a relationship with Polyna and breaks the ladder. However, we find that the ladder persists, and Tolik will still be climbing it every then and now — after travelling to Ukraine again, bringing to the Eastern frontier another much-needed car; after having a sort of breakup with Polyna, and after the couple makes up and Polyna leaves for Spain to be with him permanently:

After all, the ladder became a constant reminder for Tolik of the kind of self he no longer wanted to inhabit. And yet, even after that, he kept climbing the ladder. ( 14 on page 276, penultimate lines)

The “past Tolik” — unstraightened, odd, “queer” — thus keeps haunting the seemingly “normalized” Tolik who has disidentified with his “previous self” and now follows the “straight line” more or less.

*The Ladder* is left open to interpretations of what exactly has caused Tolik’s “straightening” and abandoning his queer ways. Heteronormative reading of the novel would lead us to believe that, first, Tolik was affected by the discrepancy in the attitudes towards Russians between him and his European colleagues, the latters’ sympathy towards “nice, oppositionary Russians” prompting the protagonist to bond with his kin who understandably do not tolerate any Russians at all — and, second, that Tolik somehow has “naturally” fallen in romantic love with Polyna. Nevertheless, it is difficult to call a romantic plotline detailed or convincing enough. Tolik only retreats to Polyna after his previous vague love interest, Alba, chooses an “oppositionary Russian” over him. The reader barely gets any insights into Tolik’s feelings towards Polyna apart from not wanting her to leave, in a very similar way to him, not wanting the rest of the family to leave. Polyna does not seem to be excited by Tolik at any point as well; there are very few hints that she might have more than simply tolerated his company. The only wish that Polyna explicitly voiced was to get back to her apartment in Ukraine, once that part of the country would be deoccupied, and never leave it again, to “spread the roots till the magma layer in the Earth’s core” ( 14 on page 50). Apart from that, she shares with Irusya not feeling or wanting anything at all ( 14 on page 50).

In fact, it is Tolik’s aunt, Hryhorivna, who almost manipulates the two to get together by employing “reverse psychology,” hinting that one might have feelings for the other, and even outright lying:

With every bolt screwed into the [Ford] Raptor, Hryhorivna screwed her own attitudes into Polyna and Tolik.
— Daughter, — she said to Polyna, — Tolik said he’s about to go to Ukraine.
— He didn’t tell me that.
— He said he wouldn’t leave you.
Polyna stayed silent.
On the way back to Vladik that day, Hryhorivna told Tolik that Polyna said: “If only he would go with me!.”
— Did she actually put it like that? — asked Tolik.
— Tolya-Tolya, — Hryhorivna shook her head. — I am a woman. A woman would only trust such words to another woman! How do you imagine she’d said that to you? ( 14 on page 269)

The whole relationship is thus largely facilitated by Tolik’s elders — a prime example of Ahmed’s “straightening devices” at work. Another important “straightening device” affecting Tolik is shame. He experiences a lot of shame as a Ukrainian man who is not fighting, especially after his cousin’s death. While Tolik seems to have recognised and done all he could to escape if not eliminate his family’s preconceptions concerning “proper” masculinity, he accepts without a question the larger, nationwide gendered normativity discourse requiring every “proper” male member of the nationhood to quit everything and fight the enemies ^28, 29^.

Ahmed notices that sometimes escaping one “straight line” means little but following the other. Particularly in the case of queer people, various homonormative ^17^ “straight lines” have emerged. A queer individual can opt to follow such a line in order to still be considered “fitting” — for instance, by making a gay marriage working by the same measures as a “conservative” heterosexual marriage ( 8 on page 172–173). *The Ladder*’s protagonist Tolik, while escaping his family’s heteronormative “straight line,” ends up picking a “straight line” of a “successful third-country national who has migrated to Western Europe”: “fully integrated,” having a decent job, a permanent residency, and a house.

Throughout the novel, Tolik gets to conflictingly oscillate between an “immigrant’s straight line,” in which he thought to have succeeded, and the one drawn anew for Ukrainians by the war — while still trying to resist the one his family is trying to inflict upon him. The “immigrant’s straight line,” until recently, largely coincided with one of the less spoken “straight lines” presented as a success within Ukrainian society — of “successfully” moving “to the West.” The “new,” wartime Ukrainian “straight line” completely redefines how one should act and where and when one should depend on their gender. Tolik’s conflict between “the lines” becomes particularly acute after Slavka’s death:

He couldn’t make his mind up around the fact that the lispy kid behind the curtain grew up, put on the military uniform, and died. Now he was a hero, and Tolik was sitting and talking to a stone frog. Tolik was used to feeling a winner — he left his mother’s house early, studied well, won the contests, received a scholarship, has found a good job early. He was only a loser because his mom considered him unrepentant.
…
“Slavka is a hero” — thought Tolik. — “And I am a loser”. ( 14 on page 109–110)

In the end, Tolik somehow settles “in between” the lines, as much as he can: while continuing to follow his path of a “relatively successful migrant in the EU,” he contributes to the Ukrainian fight as much as possible by bringing military aid; his relationship with Polyna could be deemed satisfactory for both “straight lines.” Thus, the novel also reflects the contextual specificity and multiplicity of existing “straight lines” that might be countering each other, and that become particularly inescapable for Muñoz’s “minoritarian subject” ^9^, such as a migrant.

*The Ladder* is not only an “eccentric” ^5^ story of an adult man having to endure his relatives’ presence due to the circumstances that neither of them ever wished for. It is the story of a Ukrainian man trying to navigate and negotiate multiple, changing, diverging normativities of gender, belonging, nationhood, and how one must inhabit time and space accordingly. In this reading, his relatives are first and foremost facilitators and transmitters of these normativities. Their physical presence and the way they change Tolik’s space is, indeed, crucial for their “work” to succeed, and lead Tolik’s “great escape” to fail. As mentioned above, the novel’s critics pointed out that characters lacked transformations and development ^5^; however, this is not true if we assess the novel from a queer perspective. Tolik has indeed changed: he is, by all means, “straightened” now.

## Concluding thoughts

As obvious as it might sound, Russia’s war against Ukraine has indeed changed everything for Ukrainians: not only their surroundings, those familiar since forever no longer being safe, not only the social roles, aims, and ways of life — but the very ways of being oneself. What was unimaginable before became mundane; what was mundane too often no longer imaginable. However, none of these changes are processed, acknowledged, and conceptualised by default: it takes a lot of effort from multiple scholars, researchers, activists, and artists to connect the dots and get a more or less complete picture of what has the war done to society and individuals — just before everything would change again. Fiction literature is a great way to capture these changes in a moment, and it leaves lasting material to study further, particularly when it comes to such a reflective novel as *The Ladder* by Eugenia Kuznetsova.

In this article, I briefly outline some gendered and queer moments that I find illustrative in *The Ladder*. The topic of gender and queerness in Ukrainian society during the war is as important and complicated as it is yet underexplored — particularly, as reflected in recent Ukrainian literature. I hope that more research and analysis of wartime Ukrainian fiction literature employing gender and queer prism will follow and contribute to a better understanding of how war has changed normativities and experiencing of gender and sexuality for Ukrainians.

## Ethics and consent

Ethical approval and consent were not required.

## Data Availability

No data associated with this article.

## Footnotes

^1^ While female Ukrainian citizens mostly retain freedom of movement and action, most of the male citizens aged 18–60 are banned from leaving the country and are a subject to military draft.

^2^ Shortened version for Anatolii: normally would be used for a child or a very young man.

^3^ Following Halberstam’s “queer time” ^15^.

^4^ See also 16, for a somewhat similar argument on the “queerness of the post-socialist condition”.

^5^ Hereafter Ukrainian-English translations of the excerpts from *The Ladder* are made by the author of the article.

^6^ A patronym from “Hryhorii”, a Ukrainian male name having common origins with Gregory. Patronyms are commonly used in Ukrainian society for elders and superiors, although addressing a person with a patronym only, in separation from the name, is a rather informal greeting. In *The Ladder*, Tolik’s aunt is addressed solely with her patronym, whereas his uncle, Anatoly (name) Stepanovych (patronym), gets the full version of his name.

^7^ See 19; 20, on Western European homonationalism.

^8^ The novel doesn’t specify which exactly comments were meant, yet it is likely that Irusya was working on dismantling Russian propaganda and disinformation against Ukrainians in social media: this was one of the common “cyber volunteering” activities in the very beginning of the full-scale invasion, on par with reporting Russian propagandist accounts and channels.
