# Supplementary material for: The Ladder by Eugenia Kuznetsova: A queer reading of a wartime Ukrainian novel
Source: Open Res Eur. 2025 Aug 8;5:238. [Version 1] doi: 10.12688/openreseurope.20792.1 (PMC12411837; doi:10.12688/openreseurope.20792.1)
Supplement: Peer review history

# Reviewer report: Reviewer response for version 1

**DOI:** 10.21956/openreseurope.22496.r58098

**Published:** 2025-09-04

**Authors:** Jędrzej Burszta

**Affiliations:** 1 University of Warsa, Warsaw, Poland

The article proposes a very thought-engaging queer reading of the novel The Ladder written by Eugenia Kuznetsova. The author skillfully employs Ahmed's and Munoz's theories to broaden the understanding of "queerness" beyond its usual relation to non-normative sexuality, instead opting for a perspective aligning queer ways of life with politics, nationhood, and wartime social life. The analysis is well done and introduces new insights into both the field of Ukrainian contemporary literature as well as queer studies; it would, however, benefit from some reflection on the relationship between queerness and Ukrainian nationhood in the past and in the present, and perhaps comment on the ways in which Ukrainian subjects - queer and/or queer-ed, like the protagonist - find themselves split between Western and Eastern European notions of identity, nation, state, all in reference to queer politics and world-making addressed in the essay. Some context for queer reading of Ukrainian contemporary literature would be also warranted - e.g. is Kuznetsova's novel an outlier, and does this queer intervention align with any other recent novels?

If any, are all the source data and materials underlying the results available?

Yes

Is the work original in terms of material and argument?

Yes

Is the work clearly and cogently presented?

Yes

Is the argument persuasive and supported by evidence?

Yes

Does the research article contribute to the cultural, historical, social understanding of the field?

Yes

Does it sufficiently engage with relevant methodologies and secondary literature on the topic?

Yes

Reviewer Expertise:

queer theory, socialism, anthropology of memory

I confirm that I have read this submission and believe that I have an appropriate level of expertise to confirm that it is of an acceptable scientific standard.

# Reviewer report: Reviewer response for version 1

**DOI:** 10.21956/openreseurope.22496.r58103

**Published:** 2025-08-30

**Authors:** John paolo Sarce

**Affiliations:** 1 Ateneo de Manila University, Quezon City, Metro Manila, Philippines

For the theory, the novel is written by Ukrainian author, I wonder if it's possible to add the background of queer culture in Ukraine. Maybe add a section on Ukrainian queer culture such as key concepts or terms which can also be operated as a lens to add value to the analysis. 

 Also, add a section on method which is close reading or queer reading of the text.

If any, are all the source data and materials underlying the results available?

Yes

Is the work original in terms of material and argument?

Yes

Is the work clearly and cogently presented?

Yes

Is the argument persuasive and supported by evidence?

Yes

Does the research article contribute to the cultural, historical, social understanding of the field?

Yes

Does it sufficiently engage with relevant methodologies and secondary literature on the topic?

Yes

Reviewer Expertise:

Queer studies, Literary and cultural studies, medical humanities, and digital humanities.

I confirm that I have read this submission and believe that I have an appropriate level of expertise to confirm that it is of an acceptable scientific standard.
